# Galactomannan Downregulates the Inflammation Responses in Human Macrophages via NF*κ*B2/p100

**DOI:** 10.1155/2015/942517

**Published:** 2015-09-09

**Authors:** Víctor Toledano, Enrique Hernández-Jiménez, Carolina Cubillos-Zapata, Marta Flandez, Enrique Álvarez, Aníbal Varela-Serrano, Ramón Cantero, Gema Valles, Francisco García-Rio, Eduardo López-Collazo

**Affiliations:** ^1^Tumor Immunology Laboratory, IdiPAZ, La Paz Hospital, 28046 Madrid, Spain; ^2^Innate Immunity Group, IdiPAZ, La Paz Hospital, 28046 Madrid, Spain; ^3^EMPIREO S.L., 28004 Madrid, Spain; ^4^CIBER-BBN, 28029 Madrid, Spain; ^5^Respiratory Disease, IdiPAZ, La Paz Hospital, 28046 Madrid, Spain

## Abstract

We show that galactomannan, a polysaccharide consisting of a mannose backbone with galactose side groups present on the cell wall of several fungi, induces a reprogramming of the inflammatory response in human macrophages through dectin-1 receptor. The nuclear factor kappa-light-chain-enhancer of activated B cells 2 (NF*κ*B2)/p100 was overexpressed after galactomannan challenge. Knocking down NF*κ*B2/p100 using small interfering RNA (siRNA) indicated that NF*κ*B2/p100 expression is a crucial factor in the progression of the galactomannan-induced refractoriness. The data presented in this study could be used as a modulator of inflammatory response in clinical situations where refractory state is required.

## 1. Introduction

Galactomannan (GAL) is a component of the cell wall of several fungi and is released during growth [1]. Detection of GAL in blood is used to diagnose invasive fungal infections [2, 3]. Several studies have demonstrated invasion by fungi occurring primarily in the setting of immunosuppression or during refractory states [4, 5]. Septic patients, with normal immune function prior Intensive Care Unit admission, developed a refractory status and, subsequently, an invasive pulmonary aspergillosis [6]. We have generalized a refractory state caused by the presence of bacteria which may lead to the invasion of fungal infections. However, there are not data on the potential effect of fungi invasion on the modulation of the innate immune response.

We and others have described that lipopolysaccharide (LPS), the major component of the outer membrane of Gram-negative bacteria, causes monocytes/macrophages to enter into a transient state in which they are refractory to further endotoxin stimulation [7, 8]. This phenomenon has been described in several clinical situations such as sepsis where circulating cells isolated from patients show a reduced capacity to produce proinflammatory cytokines when stimulated with an endotoxin challenge [9, 10]. Similar data has been reported for other pathologies such as acute coronary syndrome [11], cystic fibrosis [12–14], and chronic lymphocytic leukemia [15]. Previous studies from our group and others had demonstrated the involvement of the nuclear factor kappa-light-chain-enhancer of activated B cells 2 (NF*κ*B2)/p100 in this phenomenon [16, 17].

Here, we studied the effect of GAL on the innate response. We hypothesized that GAL would induce a macrophage reprogramming, leading to a refractory state. This hypothesis was tested using an* in vitro* model of human macrophages.

## 2. Materials and Methods

### 2.1. Reagents

The following antibodies were used: anti-p100/p52 and anti-*β*-actin (cell signaling). The LPS from* Salmonella abortus* was a kind gift from Dr. Galanos (Max Planck Insitut für Immunobiologie, Freiburg, Germany). D-Galacto-D-mannan was purchased from Sigma-Aldrich. The medium used for the cell culture was Dulbecco's Modified Eagle Medium (DMEM) from Invitrogen. Whole glucan particles (WGPs) soluble (1,3/1.6 *β*-glucan from* S. cerevisiae*) was used as dectin-1 inhibitor activity, purchased from Invitrogen [18, 19].

### 2.2. Isolation, Differentiation, and Culture of Human Macrophages

Mononuclear cells from peripheral blood were isolated from the buffy coats. The monocytes were obtained by Ficoll/Percoll-plus gradient (GE Healthcare Bio-Sciences), as previously described [14]. Human macrophages were obtained by differentiation of monocytes with recombinant human M-CSF-(Peprotech) at 50 ng/mL for 10 days. The purity of the macrophages was tested by CD14 labeling and flow cytometry analysis (average of 94% of CD14 positive cells). Other cell surface markers were also tested (CD64: 75%; CD11b: 95%; see Supplementary Figure  1 available online at http://dx.doi.org/10.1155/2015/942517). All the reagents used for the cell culture were endotoxin-free, as assayed with the* Limulus* amebocyte lysate test (Cambrex). The workflow to establish the different* in vitro* models was as indicated in each figure legend.

### 2.3. RNA Isolation and Quantification

The cells were washed once with PBS and the RNA was isolated using the High Pure RNA Isolation Kit (Roche Diagnostics). The cDNA was obtained by reverse transcription of 1 *μ*g of RNA using the High Capacity cDNA Reverse Transcription Kit (Applied Biosystems).

Gene expression levels were analyzed by real-time quantitative PCR (qPCR) using the Light Cycler system (Roche Diagnostics) and cDNA obtained as described above. qPCRs were performed using the QuantiMix Easy SYG kit from Biotools and specific primers. Results were normalized to the expression of the *β*-actin (actin), and the cDNA copy number of each gene of interest was determined using a 7-point standard curve as described previously [10, 11, 13, 20, 21]. The products were amplified using primers for CIITA, 5′-ATT TGG CAG CAC GTG GTA CAG GA-3′ (forward) and 5′-TCT GCA CAA GCT TTC CCA GG TCT T-3′ (reverse), and actin, 5′-GTG GGG CGC CCC AGG CAC CA-3′ (forward) and 5′-CTC CTT AAT GTC ACG CAC GAT TTC-3′ (reverse). All primers were synthesized, desalted, and purified by Bonsai Biotech.

### 2.4. Flow Cytometer Analysis

For the surface marker staining, the cells were labeled with the following monoclonal antibodies: anti-CD14 (Immunostep), anti-CD11b, and anti-CD64 (BD) and HLA-DR (Immunostep). Matched isotype antibodies were used as negative controls. The cells were incubated in the dark for 30 min at 4°C. The data was analyzed by flow cytometry using a BD FACSCalibur flow cytometer (BD Biosciences). The data was analyzed with Cell Quest Pro software (BD Bioscience).

### 2.5. Proliferation Assay

We followed a protocol described by Jurado-Camino et al. [15].

### 2.6. Cytometric Bead Array (CBA)

The cytokine levels in the culture supernatants from the human samples were determined using the CBA Flex Set (BD Biosciences) following the manufacturer's protocol. The data collected were analyzed by flow cytometry using a BD FACSCalibur flow cytometer (BD Biosciences).

### 2.7. Western Blot

The monocyte cultures were harvested and washed with ice-cold PBS containing 2 mM phenylmethanesulfonyl fluoride and 2 mM CaCl_2_ (pH 7.4). Then, they were lysed by sonication (Microson Heat System) in a solubilization buffer containing protease inhibitors (200 *μ*g/mL soybean trypsin inhibitor, 1 mg/mL benzamidine, 1 mg/mL aminocaproic acid, and 2 mM phenylmethanesulfonyl fluoride) and phosphatase inhibitors (20 mM Na_4_P_2_O_7_ and 100 mM NaF).

Proteins were measured in aliquots of cell lysates using the Bio-Rad protein assay. Briefly, proteins were resolved in 8% SDS-PAGE. Gels were then blotted onto nitrocellulose and electrotransferred. Blots showing lanes with equal amounts of proteins were incubated with 5% nonfat milk in Tris buffered saline (TBS; pH 7.4) for 30 min at room temperature. Blots were then incubated overnight at 4°C with antibodies diluted in 5% nonfat milk in TBS. Antibodies used for Western blots were rabbit anti-NF*κ*B2 p100/p52 and anti-IRAK-M (cell signaling) *β*-actin (Santa Cruz Biotechnology, Inc.). Blots were then rinsed repeatedly in TBS and incubated for 1 h at room temperature with alkaline phosphatase-conjugated goat anti-rabbit IgG or goat anti-mouse IgG secondary antibodies diluted in 5% nonfat milk in TBS. After rinsing with TBS, blots were incubated with the alkaline phosphatase substrate (5-bromo-4-chloro-3-indolyl phosphate/nitroblue tetrazolium tablets; Sigma-Aldrich).

### 2.8. Small Interfering RNA (siRNA)

Life Technologies designed and synthesized the NF*κ*B2 (p100) and the control siRNAs. The monocytes were transfected with siRNAs using the Amaxa Nucleofector system (Amaxa Biosystems). Briefly, 1 × 10^6^ monocytes, mixed with 25 *μ*M of siRNA in 100 *μ*L of transfection buffer, were transferred to an electroporation cuvette and nucleofected according to the manufacturer's instructions. The cells were then immediately transferred into a 6-well culture plate (Costar) containing 2 mL of prewarmed RPMI medium supplemented with 10% heat-inactivated fetal bovine serum (FBS; Invitrogen). The nucleofected cells were cultured at 37°C with 5% CO_2_ for 1 h before the assays.

### 2.9. Statistical Analysis

The number of experiments analyzed is indicated in each figure legend. The data were collected from a minimum of three experiments and are expressed as mean ± SD. The statistical significance was calculated using paired *t*-test. The statistical significance was set at *p* < 0.05 and all statistical analyses were conducted using Prism 5.0 software (GraphPad).

## 3. Results

### 3.1. Galactomannan (GAL) Inhibited the LPS-Induced Inflammation in Human Macrophages

**Figure 1:**
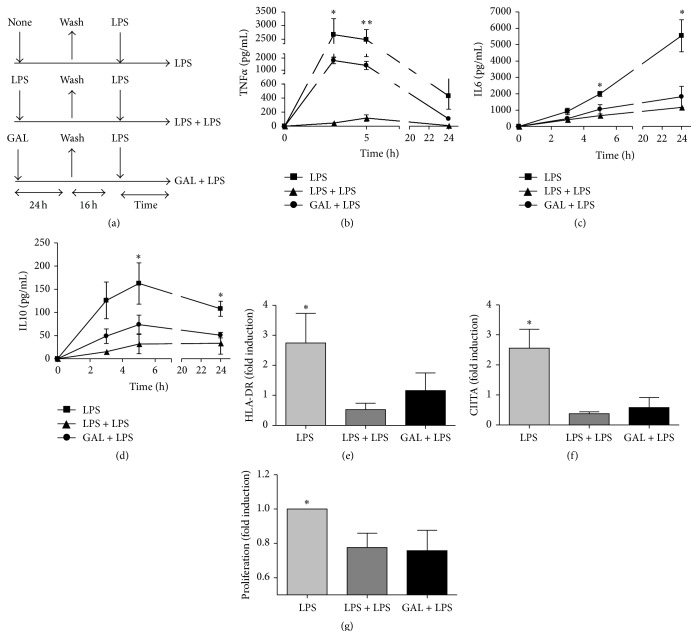
Galactomannan (GAL) attenuates the LPS-induced inflammation in human macrophages. (a) Schematic representation of the ET model used for this study. The cultures of human macrophages were or not pretreated with 10 ng/mL LPS or 10 *μ*g/mL GAL for 24 h, washed twice with PBS, cultured in medium for 16 h, and restimulated with 10 ng/mL LPS for the indicated times. Supernatants were harvested at the indicated times (3 h, 5 h, and 24 h) and TNF*α* (b), IL6 (c), and IL10 (d) proteins levels were evaluated by CBA (*n* = 5); ^*∗*^ *p* < 0.05, ^*∗∗*^ *p* < 0.01 comparing GAL + LPS with LPS. (e) Cell surface expression of HLA-DR was analyzed by flow cytometry. (*n* = 5), ^*∗*^ *p* < 0.05, comparing GAL  +  LPS with LPS 24 h. (f) Total mRNA was isolated from cells, cDNA was synthesized and CIITA real-time quantitative PCR was performed. (*n* = 4), ^*∗*^ *p* < 0.05 comparing GAL + LPS with LPS 24 h. (g) Human heterologous lymphocytes were labeled with the membrane stain PKH2 Green Fluorescent Cell Linker Kit (Sigma-Aldrich) and cocultured with LPS, LPS + LPS, or GAL + LPS macrophages for 5 days. Lymphocyte proliferation was measured as a loss of green fluorescence intensity in the CD3^+^ gate, for this analysis. The fold induction is shown. ^*∗*^ *p* < 0.05, comparing GAL + LPS with LPS.

It is well accepted that human monocytes and macrophages (MΦs) preconditioned with endotoxins, mitochondrial Danger Associated Molecular Patterns (DAMPs), or some tumor cells are unable to orchestrate a classic inflammatory response once they are challenged with LPS [8, 14, 15, 22]. To study the potential role of GAL as regulator of inflammation, human MΦs were exposed to 10 *μ*g of GALfor 24 h [4]. Next, cultures were washed, maintained in complete medium, and challenged with 10 ng/mL of LPS (see scheme in Figure 1(a) and cell purity in Supplemental Figure  1A). Note that, GAL was checked by LAL (*Limulus amebocyte lysate*) test to rule out endotoxin contamination (data not shown). As LPS prestimulation, GAL impeded the production of proinflammatory cytokines in a second stimulation with LPS (Figures 1(b) and 1(c)). Remarkably, IL10, a well-known anti-inflammatory cytokine, is also downregulated (Figure 1(d)). The expression and regulation of IL10 are controversial in this context. Several authors have shown that IL10 plays a crucial role in ET control, whereas others have reported a weak effect of IL10 in sepsis-induced tolerance [8]. In addition, IL10 knockout mice reproduced an ET phenotype when they were exposed twice to LPS [23]. Another feature of ET, such as HLA downregulation, was also observed (Figure 1(e)). In addition, GAL pretreatment downregulated the expression of HLA-DR. In this case, we also confirmed a patent reduction of the HLA-II master regulator gene, CIITA (Figure 1(f)). Finally, we verified the functional impact of MHC class II downregulation via a lymphocyte-proliferation assay (Figure 1(g)). The inhibitory effect is abolished after 5 days (Supplemental Figure  2).

### 3.2. Galactomannan Did Not Induce an Inflammatory Response in Human Macrophages

**Figure 2:**
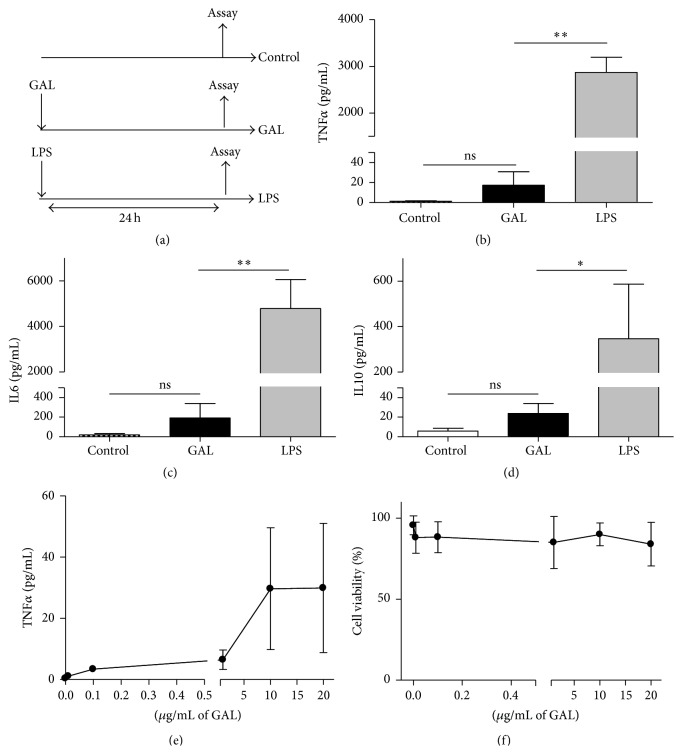
GAL is not able to induce an inflammatory response. (a) Schematic representation of the model used for this study. The cultures of human macrophages were treated with 10 ng/mL LPS or 10 *μ*g/mL GAL for 24 h. The controls were not treated. Supernatants were harvested and TNF*α* (b), IL6 (c), and IL-10 (d) proteins levels were evaluated by CBA (*n* = 6); ^*∗*^ *p* < 0.05, ^*∗∗*^ *p* < 0.01 compared with LPS. (e) The culture of human macrophages was treated with GAL at various concentrations for 24 h. Supernatants were harvested and the TNF*α* protein levels were evaluated by CBA (*n* = 3). (f) The culture of human macrophages was treated with GAL at various concentrations for 24 h. Cells were harvested, and intracellular cells stained with 7AAD were analyzed by flow cytometry. The 7AAD negative cells are represented (percentage of cell viability, *n* = 4).

Once we established that GAL induces a refractoriness state in human MΦs, we sought to understand the bases of this phenomenon. We test the effect of GAL on cultures of humans MΦs (see the experimental design in Figure 2(a)). GAL did not provoke a strong inflammatory response in these cells (Figures 2(b)–2(d)). Remarkably, mitochondrial DAMPs, such as mtDNA, do not induce a manifest inflammatory response in human monocytes but rather a refractory status [24]. Notably, an increase in the dose of GAL had no significant effect on the production of TNF*α* and other cytokines (Figure 2(e) and data not shown), and cell viability was not affected (Figure 2(f)).

### 3.3. NF*κ*B2/p100 Is Overexpressed in Human Macrophages after Galactomannan Challenge

**Figure 3:**
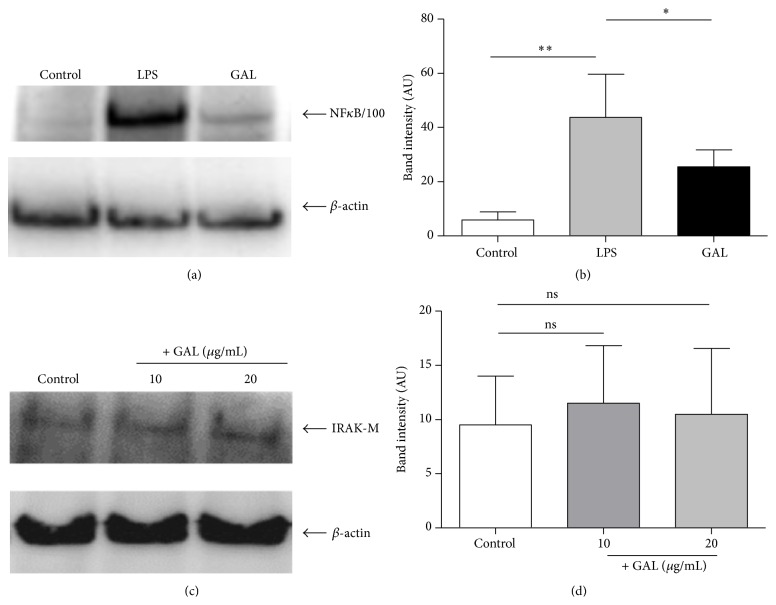
The NF*κ*B2/p100 accumulation after the first LPS or GAL challenge. Cultures of human macrophages were challenged with 10 ng/mL LPS or 10 *μ*g/mL GAL for 24 h. The controls were not treated. The levels of NF*κ*B2/p100 or IRAK-M and *β*-actin were studied by Western blot analysis of the cytosolic fraction. (a) A NF*κ*B2/p100 standard blot is shown (*n* = 6). (b) Densitometry analysis: arbitrary units [AU] of NF*κ*B2/p100 bands are normalized with respect to *β*-actin (*n* = 6). ^*∗*^ *p* < 0.05, ^*∗∗*^ *p* < 0.01 compared with LPS. (c) A IRAK-M standard blot is shown (*n* = 3). (d) Densitometry analysis: arbitrary units [AU] of IRAK-M bands are normalized with respect to *β*-actin (*n* = 3).

A number of previous studies indicate that disruption of the classical NF*κ*B2 pathway is closely involved in the control of inflammation [7]. Many molecules have been studied and postulated to be the main regulators in this regard such as IRAK-M and, more recently, NF*κ*B2/p100 [11, 25, 26]. However, in our model only NF*κ*B2/p100 was overexpressed after GAL challenge (Figures 3(a) and 3(b)). In contrast to NF*κ*B2/p100, the Western blot analysis indicated that GAL did not increase the basal levels of the pseudokinase IRAK-M (Figures 3(c) and 3(d)).

### 3.4. Dectin-1 Mediates Macrophage Recognition of GAL

**Figure 4:**
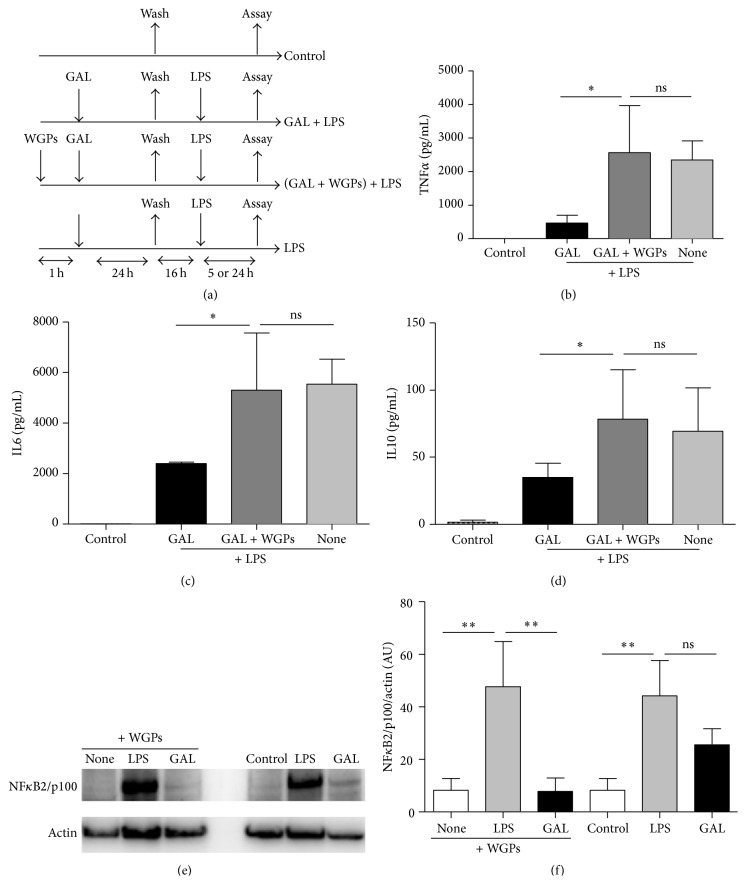
GAL is mediated by dectin-1 receptor. (a) Schematic representation of the inhibitor receptor of GAL model used for this study. The cultures of human macrophages were pretreated with or without WGPs (dectin-1 receptor inhibitor) 200 *μ*g/mL for 1 hour. Next, GAL was added to the cells for 24 h, and then cells were washed three times and cultured in resting for 16 h. Cells were challenged with 10 ng/mL LPS for 5 h or 24 h. The controls were not pretreated. Supernatants were harvested at 5 h for TNF*α* (b), 24 h for IL6 (c), and 5 h for IL10 (d). Proteins levels were evaluated by CBA (*n* = 4); ^*∗*^ *p* < 0.05 compared with LPS. Levels of NF*κ*B2/p100 and *β*-actin were studied by Western blot analysis of the cytosolic fraction. (e) A NF*κ*B2/p100 standard blot is shown (*n* = 3). (f) Densitometry analysis: arbitrary units [AU] of NF*κ*B2/p100 bands are normalized with respect to *β*-actin (*n* = 3). ^*∗∗*^ *p* < 0.01 compared with LPS.

Dectin-1 is mainly membrane-bound receptor that recognizes polysaccharide structures. We hypothesized that GAL might be recognized by dectin-1. A well-known dectin-1 receptor inhibitor (WGPs) was added simultaneously with GAL (Figure 4(a)). GAL impeded the production of proinflammatory cytokines in a second stimulation with LPS whereas the WGPs revert this tolerant state (Figures 4(b)–4(d)). In addition, NF*κ*B2/p100 is not overexpressed using the WGPs only in GAL stimulation (Figures 4(e) and 4(f)). This data could indicate that dectin-1 modulates TLR pathway by NF*κ*B2/p100. A collaborative induction and trigger modulations of inflammatory responses by these two pathways are reported [27–30].

### 3.5. Specific NF*κ*B2/p100 Downregulation Reverts the Refractoriness Induced by Galactomannan

**Figure 5:**
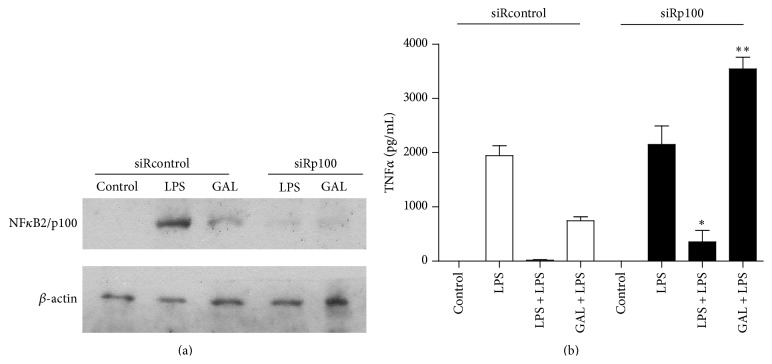
The attenuated inflammation effect induced by GAL is reverted by small interfering RNA of NF*κ*B2/p100. Cultures of siRp100 and sirRcontrol transfected human monocyte/macrophages were treated with 10 g/mL GAL or 10 ng/mL LPS for 24 h. The controls were not treated. The levels of NF*κ*B2/p100 and *β*-actin were studied by Western blot analysis of the cytosolic fraction. (a) A NF*κ*B2/p100 and *β*-actin standard blot are shown (*n* = 5). (b) TNF protein level was evaluated by CBA (*n* = 4); ^*∗*^ *p* < 0.05, ^*∗∗*^ *p* < 0.01 compared with siRcontrol.

To study the impact of NF*κ*B2/p100 on GAL-induced ET status, we knocked NF*κ*B2/p100 down using siRNA. The human monocytes were transfected with a siRNA for NF*κ*B2/p100 (siRp100) or siRNA control (siRcontrol) as a negative control. In siRcontrol-transfected cells, NF*κ*B2/p100 induction was observed after LPS or Gal challenge for 24 hours, whereas siRp100-transfected cells abolished the induction (Figure 5(a)). The siRp100-transfected cells recover the TNF*α* production when the cells were pretreated with LPS and GAL (Figure 5(b)). Other cytokines were tested with similar results (data not shown).

## 4. Discussion

Several previous studies have demonstrated that two consecutive LPS treatments separated in time reduced significantly the inflammatory response in the second challenge [7, 8, 14]. This phenomenon has been also described in patients that suffered from sepsis, acute coronary syndrome (ACS), and chronic lymphocytic leukemia (CLL), in which their circulating cells were unable to produce inflammation after an* ex vivo* endotoxin challenge [15, 24, 31]. While in sepsis patients were previously exposed to bacterial endotoxins, in ACS and CLL these patients were endotoxin-free. In these last pathologies mitochondrial DAMPS and tumor cells were responsible for the refractoriness observed, respectively. These data suggested that not only endotoxins are able to provoke a refractory state of the human innate immune cells. Additionally, the clinical experience in Intensive Care Units revealed a putative correlation between an invasive pulmonary* Aspergillosis* spp. and a refractory state in septic patients [6]. In this regard we explore the effect of galactomannan as a potential regulator of inflammation.

Data present here indicate that although GAL did not induce any inflammatory response, this polysaccharide is able to reprogram human monocytes and it reduces the inflammation induced by LPS. GAL affected not only cytokine production but also HLA-DR expression. This well-known MHCII member has been reported as essential for the switch to the adaptive response and it is downregulated in septic patients during the immunosuppression phase [32–35]. Moreover, we also reported a patent regulation of the master regulator CIITA [14, 36], being the reason by which HLA-DR expression was reduced.

A key role for dectin-1 in the response to some fungal infections was first demonstrated by the finding that dectin-1 knockout mice are more susceptible to infection with* Candida albicans* or* Aspergillus fumigates* [37, 38]. TLRs and dectin-1 can activate both NF*κ*B2 and MAPK cascades. However, they signal through an ITAM-like motif in their cytoplasmic domain, which recruits the tyrosine kinase Syk and RAF proto-oncogene serine/threonine-protein kinase to activate downstream signaling [39–41]. Moreover, in macrophages and dendritic cells dectin-1 and TLR are synergistic in mediating production of cytokines [30, 42]. Our data suggest that GAL recognition by dectin-1 receptor in macrophages attenuates the inflammation response.

A number of molecular mechanisms have been implicated in the control of inflammation after endotoxin challenge [7]. The activation of the pseudokinase IRAK-M has been described as essential to control the intracellular inflammatory pathway [26, 43–46]. However, our data did not indicate a role for IRAK-M in the observed GAL-induced inhibition of inflammation. In contrast, a patent expression of NF*κ*B2/p100 was detected after GAL treatment. This member of the noncanonical pathway exhibits inhibitory properties on the canonical pathway [47]. In this regard, NF*κ*B2/p100 could be considered as a fourth IkB protein, sequestering latent NF*κ*B2 dimers [47]. Our results demonstrated that after blocking the main GAL receptor, dectin-1, the expression of NF*κ*B2/p100 was also reduced. Moreover, knocking down assays indicated that the expression of NF*κ*B2/p100 was mandatory in the studied context.

In conclusion, the data presented in this study indicate that galactomannan could be used as a controller of the inflammatory responses in some clinical situations. Curiously, its effect did not involve a previous inflammation. These results indicate that the presence of GAL and, subsequently, fungi contamination could lead to a refractory state in patients. Our data also indicated that galactomannan acts through dectin-1 and NF*κ*B2/p100 plays a pivotal role in its refractory state.

## Supplementary Material

Supplementary Figure 1 shows the validation of macrophage purification used in the experiments described. Supplementary Figure 2 presents the levels of inflammatory cytokines over 5 days of the GAL model.
