# Supplementary material for: Galactomannan Downregulates the Inflammation Responses in Human Macrophages via NFκB2/p100
Source: Mediators Inflamm. 2015 Sep 9;2015:942517. doi: 10.1155/2015/942517 (PMC4579314; doi:10.1155/2015/942517)
Supplement: Supplementary file 1 — Supplementary Figure 1 shows the validation of macrophage purification used in the experiments described. Supplementary Figure 2 presents the levels of inflammatory cytokines over 5 days of the GAL model. [file 942517.f1.pdf]

Supplementary Appendix

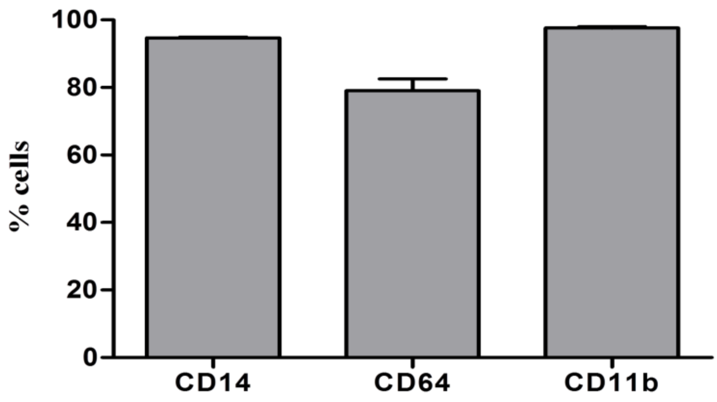

Supplementary Figure 1. Characterization of human macrophages and purity.

Human monocytes isolated and cultured with 50 ng/ml M-CSF for 10 days. Differentiated human macrophages were stained with anti-CD14, anti-CD64 and anti-CD11b. A, Percentage of CD14, CD11b and CD64 on CD14 gated cells are shown. (n=5).

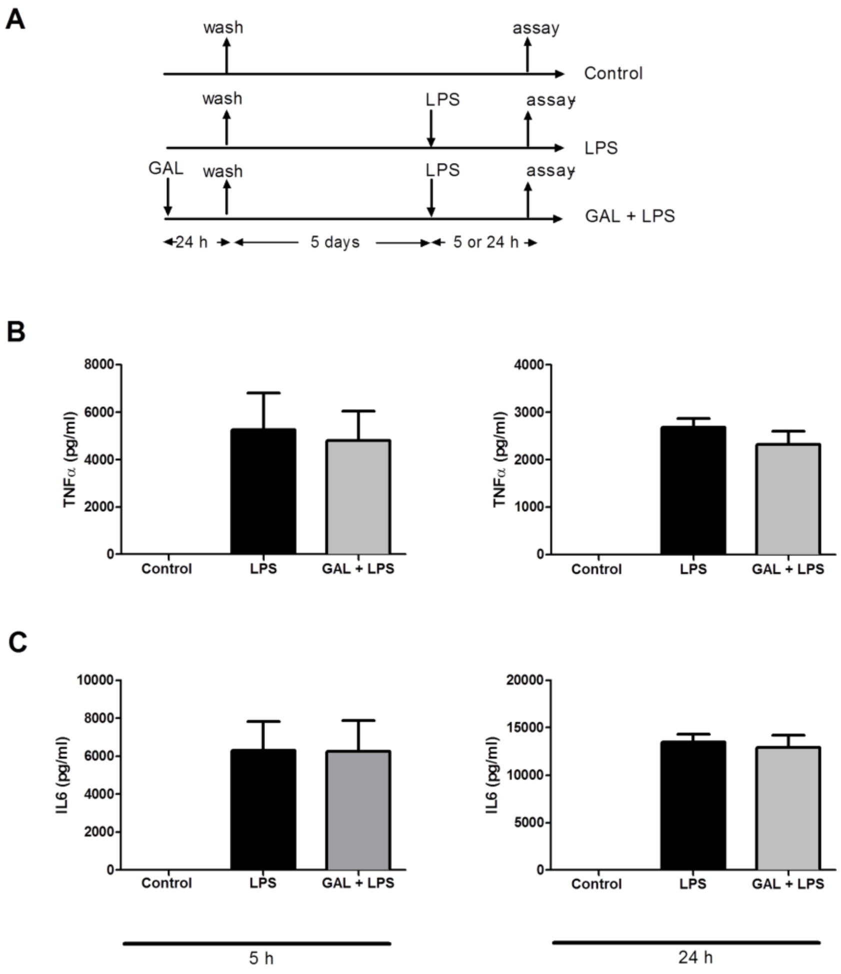

Supplementary Figure 2. GAL effects after 5 days.

A, Schematic representation of the inflammation cytokines over 5 days of the GAL model used for this study. The cultures of human macrophages were treated with 10μg/ml GAL for 24h, washed twice and then rested for 5 days. Cultured cells were challenged with 10 ng/ml of LPS for 5 or 24h. Controls were challenged or not with 10 ng/ml of LPS. Supernatants were harvested at the indicated times and TNFα (B), IL6 (C) proteins levels were evaluated by CBA; (n=4).
